# The clinical implication of soluble PD-L1 (sPD-L1) in patients with breast cancer and its biological function in regulating the function of T lymphocyte

**DOI:** 10.1007/s00262-021-02898-4

**Published:** 2021-03-10

**Authors:** Baojuan Han, Lina Dong, Jing Zhou, Yan Yang, Jiaxun Guo, Qijia Xuan, Kun Gao, Zhenguo Xu, Wanting Lei, Jingxuan Wang, Qingyuan Zhang

**Affiliations:** 1 Department of Medical Oncology, Harbin Medical University Cancer Hospital, 150 Haping Road, Harbin, 150040 People’s Republic of China; 2 Department of Radiation Oncology, The Affiliated Hospital of Qingdao University, 266000 Shandong, People’s Republic of China; 3 Department of Head and Neck Surgery, Harbin Medical University Cancer Hospital, Harbin, 150040 People’s Republic of China

**Keywords:** SPD-L1, Breast cancer, Clinical implication, Predictive marker, T lymphocyte

## Abstract

This work investigated the clinical prognostic implications and biological function of plasma soluble programmed cell death ligand 1 in breast cancer patients. Plasma sPD-L1 levels of recurrent/metastatic breast cancer patients were determined, and the association of sPD-L1 levels and metastatic progression-free survival and metastatic overall survival was assessed. The PD-L1 expression on breast cancer cells was analyzed by flow cytometry, and the level of sPD-L1 in the supernatant of breast cancer cells was determined by enzyme-linked immunosorbent assay. Furthermore, the effect of sPD-L1 on the proliferation and apoptosis of T lymphocytes was detected by WST-1 assay and flow cytometry. The plasma sPD-L1 levels in 208 patients with recurrent/metastatic breast cancer before receiving first-line rescue therapy were measured. The optimal cutoff value of plasma sPD-L1 for predicting disease progression was 8.774 ng/ml. Univariate and multivariate analyses identified high sPD-L1 level (≥ 8.774 ng/ml) and visceral metastasis were independent factors associated with poor prognosis. Relevance analysis showed that the plasma sPD-L1 level was weakly

associated with some systemic inflammation markers, including white cell count (WBC), absolute monocyte

count, and absolute neutrophil count. Furthermore**,** we found sPD-L1 could be found in supernatant of culture with breast cancer cell line expressing PD-L1 on the cell surface and inhibit T lymphocyte function, playing a negative regulatory role in cellular immunity. sPD-L1 was a good tumor predictive maker in breast cancer and it may play a potentially important role in immune tolerance.

**Supplementary information:**

The online version contains supplementary material available at 10.1007/s00262-021-02898-4.

## Introduction

Breast cancer is the most common malignancy in women and is the top cause of cancer-related death [1]. Early breast cancer can be curable, but the treatment of recurrent or metastatic breast cancer remains controversial. Early diagnosis, appropriate treatment and monitoring the treatment response during the treatment are of important prognostic significance for recurrent or metastatic breast cancer. Carcinoembryonic antigen (CEA) and breast cancer antigen (CA153) are the most widely used serum markers, which play an important role in the process of monitoring relapse or disease progression in breast cancer patients [2]. CEA and CA153 are the non-specific tumor markers, CA153 response paralleled disease in only approximately 50% of patients who receive anthracycline-based first-line treatment in prospective phrase of II and III trials [3]. Consequently, there is a need to identify biomarkers in breast cancer.

There is a growing body of evidence suggesting that cancer immune suppression and immune escape play essential roles in tumor progression [4, 5]**.** Thus, identification of the mechanisms involved in the escape of immune suppression might help to identify a novel prognostic biomarker**.** The programmed death receptor 1 (PD-1)/programmed death ligand 1 (PD-L1) pathway plays a critical role in regulating the endogenous immune response to cancer [6–9]. PD-1, an inhibitory immune checkpoint receptor is constitutively expressed on activated T cells, protects healthy cells from excessive inflammatory or autoimmune responses via combination with its ligands PD-L1 and PD-L2 [6, 10]. However, tumors can co-opt the PD-1/PD-L1 pathway to evade immune destruction. The binding of PD-L1 to PD-1 could inhibit T lymphocyte proliferation, cytokine production and promote T cell apoptosis, thus terminally evading the anti-tumor immune response and enabling neoplastic growth [11, 12]. Abundant studies have shown that PD-L1 is over-expressed on tumor cells and tumor-associated macrophages in multiple malignancies [6] and is negatively correlated with survival prognosis [13]. Recently, various clinical trials with anti-PD-1/PD-L1 drugs have shown improved outcomes and response rates in patients with non-small-cell lung cancer [14], gastric cancer [15], melanoma [16] and urothelial cancer [17].

Significantly, high expression of PD-L1 in tumor tissue is valuable, as is that of sPD-L1, a soluble form of PD-L1 in blood measured by enzyme-linked immunosorbent assay (ELISA) [18], which is a potential prognostic predictor in certain hematological malignancies and solid tumors [19–22]. Finkelmeier et al. [20] stated that high sPD-L1 levels could predict unfavorable outcomes in hepatocellular carcinoma patients and was positively correlated with the stages of disease. Rossille et al. [21] reported that the plasma sPD-L1 level could predict the treatment response and OS in patients with DLBCL. Similarly, in metastatic or recurrent gastric cancer, Takahashi et al. [22] also found that elevated serum sPD-L1 level was an independent predictor for poor overall survival. A study focused on immune regulatory molecules in peripheral blood mononuclear cells (PBMCs) examined PD-L1 mRNA expression in PBMCs with a significant fold change in metastatic breast cancer patients in contrast to healthy volunteers and primary breast cancer patients, indicating that PD-L1 was a specific gene related to disease progression [23]. Li Y et al. reported that serum levels of sPD-1 and sPD-L1 could be used as noninvasive biomarkers for evaluating the malignancy of TNBC before neoadjuvant chemotherapy and predicting neoadjuvant chemotherapy response in TNBC patients [24]. Nonetheless**,** the prognostic value of sPD-L1, as well as their association with clinicopathological factors in breast cancer, remains a matter of debate.

Thus, despite its potential importance, the regulatory roles, functions and biological significance of the sPD-L1 are still under investigation. Whether sPD-L1 are involved in immune regulation and disease progression of breast cancer has yet to be elucidated. It is well known that the PD-1/PD-L1 pathway is identified as the most critical mechanism of tumor evasion, inhibiting T cell proliferation, inducing T cell exhaustion and enhancing the activity of regulatory T cells. It remains to be clarified the biological function of sPD-L1 in regulating the functions of T lymphocyte in breast cancer.

In this study, the expression of sPD-L1 in the supernatant of breast cancer cell lines was detected, and the effect of sPD-L1 on the biological function of T lymphocytes in peripheral blood of healthy people was further tested. By exploring the effect of sPD-L1 on T lymphocyte function, it would provide evidence for the future use of sPD-L1 in breast cancer.

## Materials and methods

### Patients

Female patients at the Harbin Medical University Cancer Hospital who were newly diagnosed with recurrent/metastatic breast cancer between 2015 September and 2017 February were selected and consecutively recruited in the prospective study. The inclusion criteria were as follows: pathologically diagnosed breast cancer, clinical radiologically or pathologically confirmed recurrent or metastatic lesions without anti-tumor therapy since metastasis or relapse, Eastern Cooperative Oncology Group Performance Status (ECOG PS) 0 to 1, adequate hematological and organ function. Patients with a diagnosed second tumor in the previous 5 years and with severe life-threatening illness, receiving immunosuppressive medications or with HIV infection, autoimmunity disease, active infections, hematologic neoplasms, history of organ allograft and viral hepatitis were excluded. In addition, early breast cancer patients at diagnosis were enrolled as controls. A total of 208 recurrent/metastatic patients and 32 patients with preliminary confirmed diagnosis of primary early breast cancer formally entered our study after giving written informed consent for use of clinical data and materials. Two of them were lost to follow-up because of being unable to contact. This research was approved by the ethics committee of Harbin Medical University Cancer Hospital (KY2018-06).

### Treatment and radiologic evaluation

Due to its heterogeneity, breast cancer is divided into different relevant molecular subtypes using IHC. Four different molecular subtypes are categorized as follows: Lumin A-like subtype (ER/PR positive, HER2 negative, low ki67); Lumin B-like subtype (ER/PR positive, HER2 negative, high ki67); HER2 subtype; non-luminal (ER and PR negative, HER2 positive) or luminal (ER/PR positive, HER2 positive); or basal-like subtype (ER and PR and HER2 negative, namely triple-negative breast cancer). Guided by these molecular subtypes from metastatic or primary tumor biology, systemic therapy involving endocrine therapy, chemotherapy and molecular targeted therapy is the first choice for recurrent/metastatic breast cancer [25]. Patients continued to receive current first-line rescue therapy until disease progression or intolerable adverse events.

Patients were regularly radiologically evaluated every six weeks via contrast-enhanced computed tomography (CT) according to the Response Evaluation Criteria in Solid Tumors (RECIST) criteria version 1.1. The therapeutic responses were categorized as complete remission (CR), partial remission (PR), stable disease (SD), disease progression (PD) and non-evaluable (NE).

### Blood sample collection

Plasma specimens were obtained from 32 patients with early breast cancer at diagnosis, 208 recurrent or metastatic breast cancer patients at baseline, and at the evaluation time (each 2 cycles of treatment).

Blood samples were collected into lithium heparin (LH) BD blood collection vials within 4 h, centrifuged at 3500 r/min for 10 min and stored in 1000 µl aliquots at −80 °C until measurement. Experiment was repeated for three sets using the same serum samples.

Other clinical laboratory tests were examined before initiation of first-line palliative chemotherapy, including white cell count (WBC), absolute monocyte count, absolute neutrophil count, and absolute lymphocyte count. The lymphocyte-to-monocyte ratio (LMR) was calculated by dividing the lymphocyte count by the monocyte count, and the neutrophil-to-lymphocyte ratio (NLR) was calculated by dividing the neutrophil count by the lymphocyte count.

### Measurement of plasma sPD-L1

An ELISA kit was used to measure the protein concentration of sPD-L1 (PDCD1LG1 ELISA kit, USCN Life Science, Wuhan, China) in blood collected from early patient controls and recurrent/metastatic breast cancer patients. The minimum detectable dose of plasma sPD-L1 was 0.057 ng/ml. Following the manufacturer instructions, samples were measured in triplicate, and the intra- and inter-assay variations were less than 20%. The same assay and procedure were used to measure sPD-L1 levels for all studied cohorts. In our study, each sample was tested in triplicate for sPD-L1, with the medians used for analysis.

### Tissue sample collection and IHC

Biopsy of the first metastatic or recurrent lesion was proposed to verify breast cancer histology and reassess the tumor biology if clinically feasible because histology might vary from the primary site due to heterogeneity. We retrospectively 86 consecutively recruited patients with adequate metastatic tissues for PD-L1 and CD8 measurement.

PD-L1 (clone ab58810, Abcam, Paris, France) and CD8 (clone ab66868, Abcam, Paris, France) were assessed in formalin-fixed, paraffin-embedded archival tumor samples by IHC. The positive and negative controls were supplied by the manufacturer. Slides, each having 1000 tumor cells and 1000 adjacent non-tumor cells, were scored by two pathologists who did not participate in the clinical data study. Positivity was defined as PD-L1 and CD8 expression in the stroma or ≥ 1% in the tumor cells.

In this study, slides of full-face hematoxylin and eosin-stained sections from primary tumors were retrieved for the evaluation of TILs by light microscopy and we set the cutoff value at 20% and defined high TILD.

### Cell culture

Human breast cancer cell lines (MDA-MB-231, T47D, MCF-7, MDA-MB-453) were obtained from the Heilongjiang Cancer Institute (Harbin, China). MDA-MB-231 cells were cultured in L15 (Gibco, Grand Island, NY, USA) supplemented with 10% fetal bovine serum (Gibco). MCF-7and T47D cells were cultured in DMEM (Gibco, Grand Island, NY, USA) supplemented with 10% fetal bovine serum (Gibco). MDA-MB-453 cells were cultured in RPMI1640 medium (Gibco, Grand Island, NY, USA) supplemented with 10% fetal bovine serum (Gibco); both cell lines were then incubated at 37 °C in a humidified atmosphere containing 5% CO2.

### Preparation of T lymphocytes in human peripheral blood

Fresh peripheral blood of patients with heparin sodium anticoagulant was centrifuged by Ficoll density gradient centrifugation for 30 min at 1800 r/min to obtain PBMCs. The cell concentration was adjusted to 2 × 10^6^ cells/ml, further separation and purification of human T lymphocytes from PBMC using the EasySep Human Monocyte Enrichment Kit, purity > 90%; placing the cells in RPMI 1640 medium, 5% CO2, 37 ℃ were cultured to the logarithmic growth stage.

### Analysis of PD-L1 on breast cancer cell surface

Different human breast cancer cell lines were incubated with PE-mouse anti-human PD-L1 mAb (clone ab270652, Abcam, Paris, France) at 37 ℃ for 20 min. After washing with PBS, labeled cells were detected by flow cytometry and analyzed by Beckman-Coulter’s Expo32 MULTICOMP software.

### Detection of sPD-L1 in breast cancer cell culture supernatant

MDA-MB-231, T47D, MCF-7 and MDA-MB-453 cellular supernatants were centrifuged at 1000 g/min for 20 min, and the cell-free supernatants were stored at −20 °C for the ELISA assay. sPD-L1 in cell culture supernatants was measured using the PathScan total PD-L1 Sandwich ELISA Kit (Cell Signaling Technology).

### Cell viability assay

The viability of the T cells was measured by WST-1 assay. To generate activated T cells, T lymphocyte cells were treated with 10 µg/ml PHA for 3 days. The experiment was divided into 10 groups: resting T lymphocyte, activated T lymphocyte, activated T lymphocyte plus MDA-MB-231 cell supernatant, activated T lymphocyte plus T47D cell supernatant, activated T lymphocyte plus MCF-7 cell supernatant, activated T lymphocyte plus MDA-MB-453 cell supernatant, activated T lymphocyte plus MDA-MB-231 cell supernatant plus anti-PD-L1 atezolizumab (ATE 100 μg/ml), activation T lymphocyte plus T47D cell supernatant plus ATE, activated T lymphocyte plus MCF-7 cell supernatant plus ATE, activated T lymphocyte plus MDA-MB-453 cell supernatant plus ATE.

In brief, as the confluence of the MDA-MB-231, T47D, MCF-7 and MDA-MB-453 reached around 70%, they were seeded in 96-well plates. After 72-h incubation co-cultured with T lymphocyte cells, WST-1 solution (20 μL, 0.5 mg/mL in PBS) was added to each well and the plates were incubated at 37 °C for 4 h. Finally, the medium was removed and dimethyl sulfoxide (DMSO, 150μL) was added to each well for 10 min to dissolve the purple formazan crystals. Absorbance was measured at 570 nm using a microplate reader. All assays were performed in three independent experiments.

### Flow cytometry

The apoptosis of T lymphocyte was determined by flow cytometry using the Annexin V-FITC and propidium iodide (PI) staining. The experimental group was the same as above, the purified T lymphocytes were added to the 24-well culture plate at 1 × 10^6^ cells/ml, and the culture plate was placed in an incubator for 3 days, and then the cell suspension was transferred to a centrifuge tube. Then, 1 × 10^6^cells were collected and washed twice with ice-cold PBS. Cells were dual-stained using a FITC Annexin V Apoptosis Detection Kit I (BD Biosciences, Franklin Lakes, NJ) according to the manufacturer's protocol. Stained cells were immediately analyzed using a flow cytometer (BD Biosciences).

### Statistical analysis

Receiver operating characteristic (ROC) curve analysis was used to define the discriminating cutoff value with maximized sensitivity and specificity for sPD-L1 concentration.

Metastatic progression-free survival (PFS) was defined the period from newly confirmed metastatic or recurrent breast cancer to disease progression or death because of disease progression. Metastatic OS was defined the period from newly confirmed metastatic or recurrent breast cancer to death because of disease progression. Median PFS and OS were computed by the Kaplan–Meier method, and the comparisons of variance were assessed using log-rank tests. Potential survival prognostic factors for PFS and OS were assessed by univariate analysis at first, and factors with *p* value less than 0.05 were accessed to multivariate for further validation. Hazard ratio (HR) and 95% confidence interval (95% CI) for all variables were calculated in the regression model.

Comparisons of clinical data between groups were made using the χ2 test, the Mann–Whitney t-test or the Wilcoxon-matched test, as appropriate. Relevance between plasma sPD-L1 levels and other laboratory examinations was analyzed using the Spearman or Pearson correlation analysis calculating the coefficient. A two-sided verified *p* value less than 0.05 was unified as statistical significance. SPSS version 17.0 statistical software and GraphPad Prism 5.01 (GraphPad Software, La Jolla, CA, USA) were used for statistical analysis.

## Results

### Patient characteristics

A total of 208 patients with recurrent or metastatic breast cancer were enrolled in our study. The expected baseline characteristics are shown in Table 1. Among the 208 cases, according to biological tumor subtypes, 33 (15.9%) had triple-negative breast cancer, 99 (47.6%) had HER2-positive disease, and the remaining 76 (36.5%) had luminal disease. A total of 181 patients (87.0%) relapsed after surgery and received necessary adjuvant treatment according to routine clinical practice in the early-stage setting. The location of metastatic occurrence was primarily liver, lung, or both (70.7%), and the remainder were located in bone, nodal and soft tissues (29.3%).

**Table 1:** Clinical characteristics of advanced breast cancer according to optimal sPD-L1 level

Characteristics	Total patients (*n* = 208)%	Low sPD-L1 (<8.774 ng/ml)	High sPD-L1 (≥ 8.774 ng/ml)	*p* value
*Age, years*				
≤ 60	160(76.9)	98(83.8)	62(68.1)	0.005
> 60	48(23.1)	19(16.2)	29(31.9)	
*Menopause status*				
Premenopausal	104(50.0)	58(49.6)	46(50.5)	0.889
Postmenopausal	104(50.0)	59(50.4)	45(49.5)	
*IHC profile*				
Triple-negative	33(15.9)	16(13.7)	17(18.6)	0.202
HER-2-positive	99(47.6)	62(53.0)	37(40.7)	
Luminal	76(36.5)	39(33.3)	37(40.7)	
*Location of metastases*				
Visceral	147(70.7)	86(73.5)	61(67.0)	0.309
Non-visceral	61(29.3)	31(26.5)	30(33.0)	
*TNM staging*				
I-II	101(48.6)	60(51.3)	41(45.1)	0.373
III-IV	107(51.4)	57(48.7)	50(54.9)	
*Previous chemotherapy*				
Yes	159(87.8)	89(88.1)	70(87.5)	0.899
No	22(12.2)	12(11.9)	10(12.5)	
*Previous endocrine therapy*				
Yes	87(48.0)	51(50.5)	36(45.0)	0.462
No	94(52.0)	50(49.5)	44(55.0)	
*Previous radiotherapy*				
Yes	120(66.3)	59(58.4)	61(76.2)	0.012
No	61(33.7)	42(41.6)	19(23.8)	
*First-line therapy*				
Targeted therapy				0.635
Trastuzumab + taxane	84(84.8)	51(82.3)	29(60.7)	
Lapatinib + capecitabine	15(15.2)	11(17.7)	8(60.0)	
Endocrine therapy				0.589
Fulvestrant	4(17.4)	3(17.7)	1(10.0)	
AIs ± deprivation therapy	23(85.2)	14(82.3)	9(90.0)	
Chemotherapy				0.605
Capecitabine	5(5.7)	1(2.6)	4(9.1)	
Gemcitabine ± platinum	29(33.0)	13(34.2)	16(36.4)	
Vinorelbine ± platinum	7(19.3)	3(7.9)	4(9.1)	
*Taxane* ± *epirubicin/platinum*				
/capecitabine	41(46.6)	21(55.3)	20(45.4)	

Abbreviations: sPD-L1, soluble programmed death-ligand 1; IHC, immunohistochemical; AIs, aromatase inhibitors

### sPD-L1 level with clinical characteristics

**Fig. 1:**
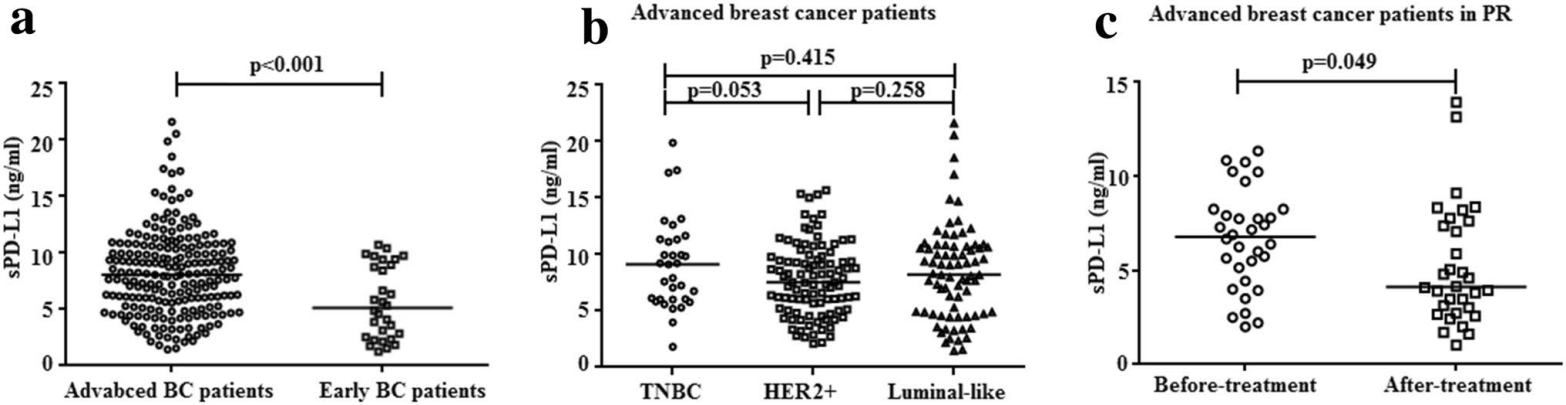
Comparison of plasma sPD-L1 level between different patients cohorts. **a** Plasma sPD-L1 protein measurement in 208 patients of our cohort before first-line treatment and 30 early breast cancer patients at diagnosis. **b** Plasma sPD-L1 protein measurement in three molecular subtypes cohort. **c** Plasma sPD-L1 protein measurement in 32 patients of our cohort in PR (partial remission) with sPD-L1 collected before first-line treatment and within 1 week after treatment. Statistical analysis was performed using the Mann–Whitney t-test or the Wilcoxon-matched test

The median sPD-L1 level in plasma collected from all recurrent/metastatic breast cancer cases before first-line rescue therapy was 7.964 ng/ml (range: 1.442–21.618), apparently higher than that of control cohorts of 30 early breast cancer patients at diagnosis (median: 4.891 ng/ml, range: 1.249–10.718, *p* < 0.001, Fig. 1a). The baseline characteristics of these early breast cancer patients are shown in Supplementary Table 1.

The optimal cutoff value of plasma sPD-L1 defined by the ROC curve in prediction of disease progression was 8.774 ng/ml (AUC = 0.676, *p* < 0.001, Supplementary Fig. 1). According to the optimal cutoff level, 91 (43.8%) patients were allocated to the high sPD-L1 (≥ 8.774 ng/ml) group, and 171 (56.2%) were allocated to the low-sPD-L1 (< 8.774 ng/ml) group. The high-sPD-L1 group were significantly associated with older age (over 60 years) and previous adjuvant radiotherapy history (*p* = 0.005 and *p* = 0.012, respectively, Table 1). In addition, the mean plasma sPD-L1 level showed no visible difference in patients with triple-negative subtype, HER2-positive subtype and luminal subtype (mean: 9.111, 7.765, and 8.402, *p* > 0.05, respectively, Fig. 1b). However, no significant correlation was noted between plasma sPD-L1 level and menopausal status, location of metastases, previous chemotherapy, endocrinotherapy history and different IHC profiles.

**Fig. 2:**
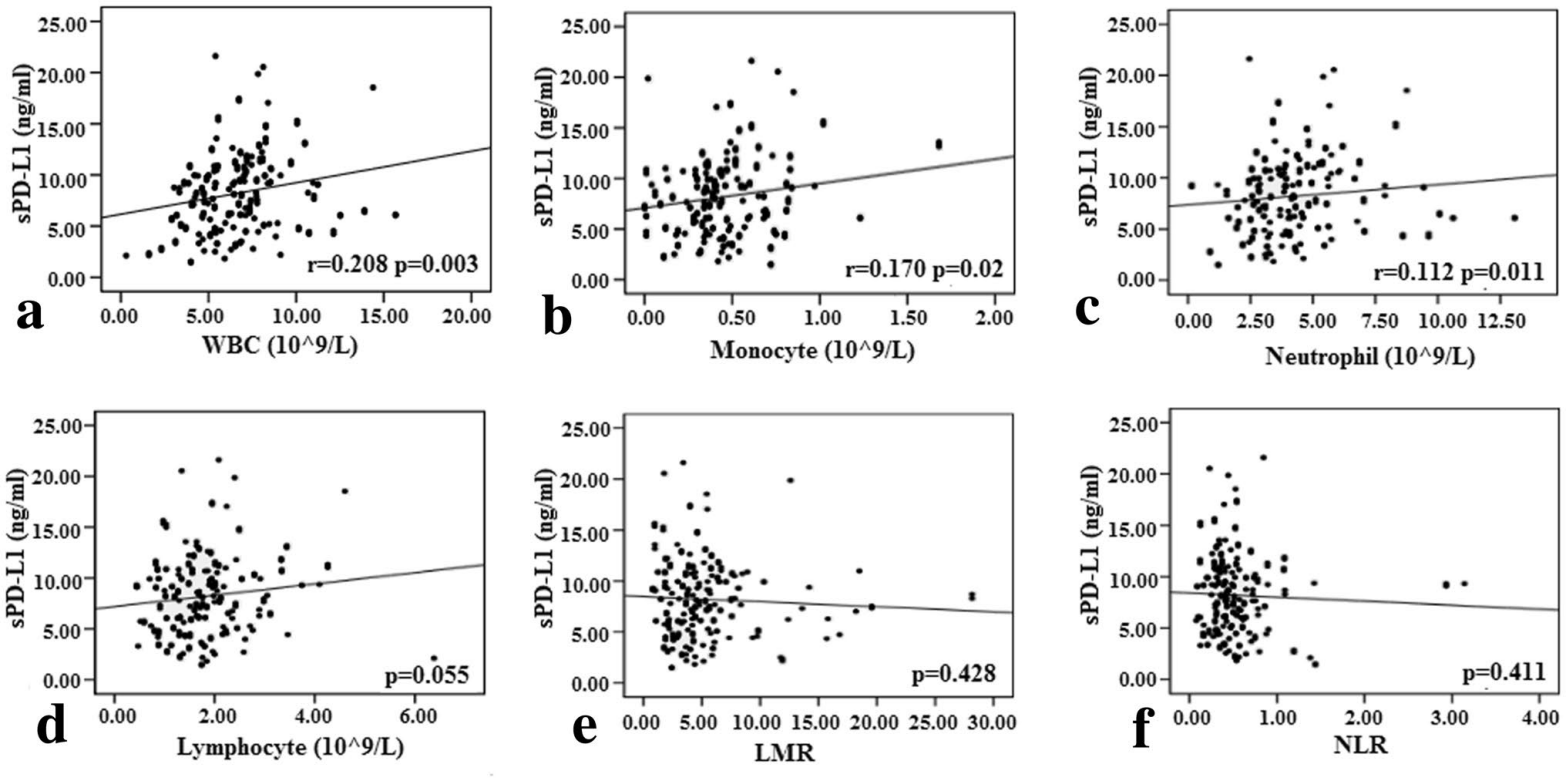
Correlation between sPD-L1 level and systemic inflammation marker. **a** WBC (white cell count), **b **monocyte count, **c** neutrophil count, **d** lymphocyte count, **e** LMR (lymphocyte-to-monocyte ratio), **f** NLR (neutrophil-to-lymphocyte). Statistical analysis was performed using the Pearson correlation **b** or Spearman correlation **b**–**f**.

In addition, the correlations of plasma sPD-L1 level with systemic inflammation markers were analyzed, including white cell count (WBC), absolute monocyte, neutrophil and lymphocyte count; lymphocyte-to-monocyte ratio (LMR); and neutrophil-to-lymphocyte ratio (NLR). Weak correlation was found between plasma sPD-L1 level and WBC (r = 0.208, *p* = 0.003, Fig. 2a), absolute monocyte count (r = 0.170, *p* = 0.020, Fig.2b), and absolute neutrophil count (*r* = 0.112, *p* = 0.011, Fig. 2c). No relevance was found for other markers, including absolute lymphocyte count, LMR and NLR (*p* = 0.055, *p* = 0.428, and *p* = 0.411, respectively, Fig. 2d-f).

### Progress analysis

**Fig. 3:**
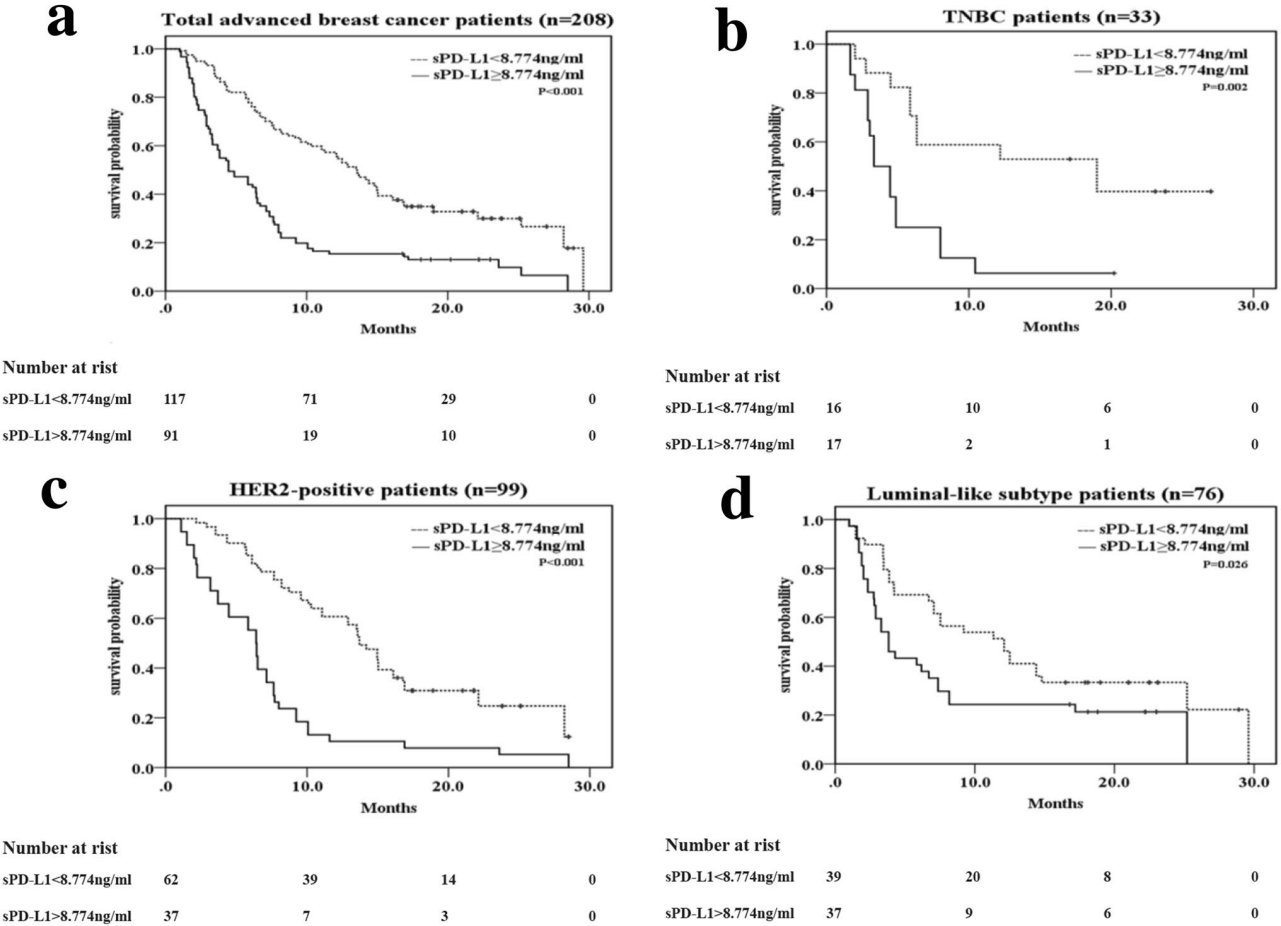
Kaplan–Meier curves of PFS for all patients and different IHC profile patients based on the cutoff sPD-L1 level (< 8.774 vs. ≥ 8.774 ng/ml). **a** All patients (*n* = 208), **b** TNBC (triple-negative breast cancer) (*n* = 33) patients, **c** HER2-positive subtype patients (*n* = 99), **d** luminal subtype patients (*n* = 76). *p* value was from Log-rank test according to the cutoff value of sPD-L1 level

**Fig. 4:**
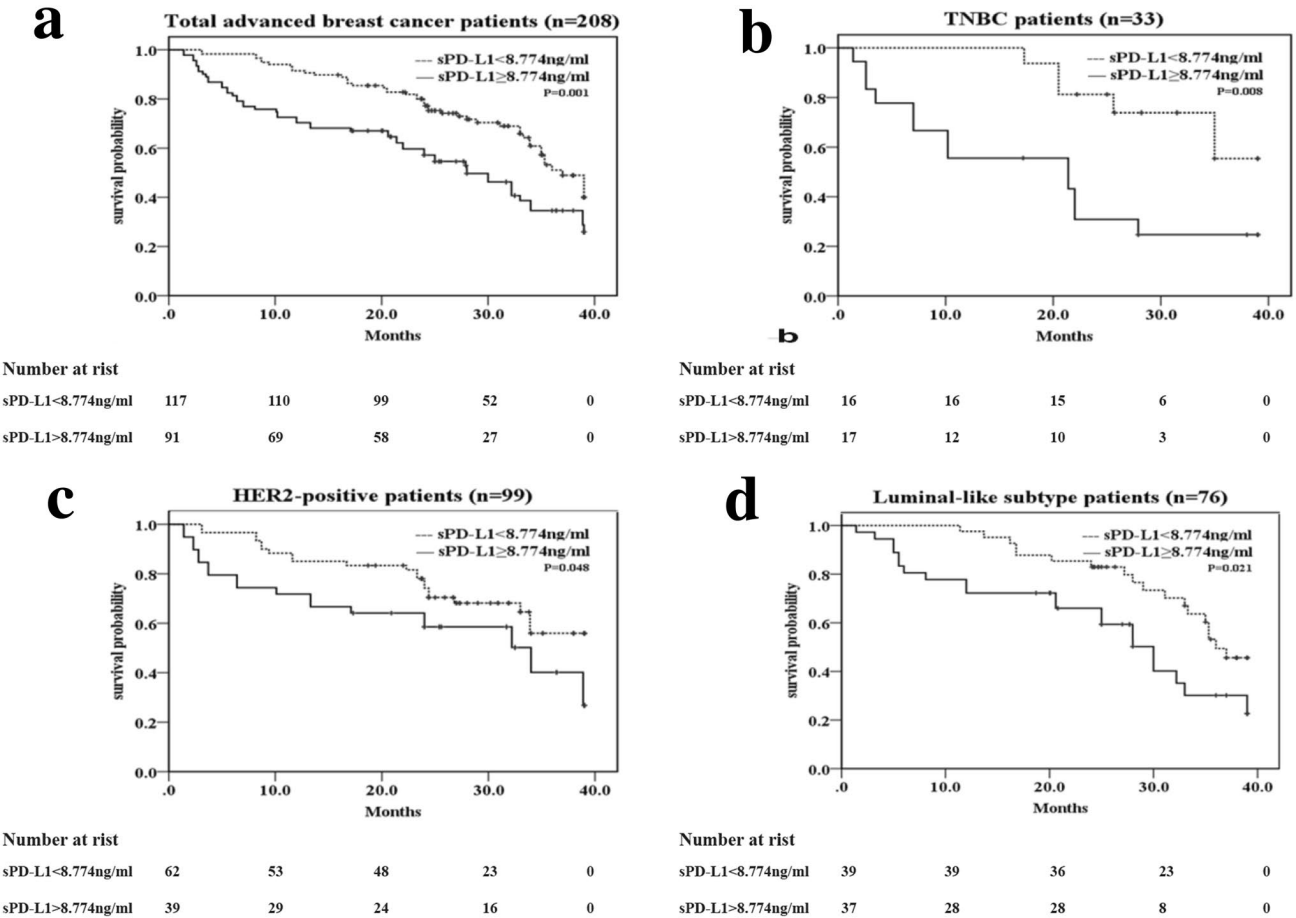
Kaplan–Meier curves of OS for all patients and different IHC profile patients based on the cutoff sPD-L1 level (< 8.774 vs. ≥ 8.774 ng/ml). ** a** All patients (*n* = 208), **b** TNBC (triple-negative breast cancer) (*n* = 33) patients, **c** HER2-positive subtype patients (*n* = 99), **d** luminal subtype patients (n = 76). *p* value was from Log-rank test according to the cutoff value of sPD-L1 level

The median follow-up was 25.2 months (range: 1.5–39.0), at which 166 (79.3%) cases exhibited disease progression and 99 patients died of breast cancer. As shown in Fig. 3, 4, high-sPD-L1 patients had poorer prognosis than low-sPD-L1 patients. Of all of the recurrent/metastatic patients, Kaplan–Meier analysis showed that patients with sPD-L1 ≥ 8.774 ng/ml had poorer PFS and OS than the patients with sPD-L1 < 8.774 ng/ml (PFS 7.2 m vs. 13.6 m, *p* < 0.001; OS 21.4 m vs 28.0 m, *p* = 0.001; Figs. 3a, 4a). For triple-negative breast cancer patients, patients with sPD-L1 ≥ 8.774 ng/ml had a significantly poorer outcome than those with sPD-L1 < 8.774 ng/ml (PFS 5.1 m vs. 13.9 m, *p* = 0.002; OS 17.4 m vs 26.6, p = 0.008; Figs. 3b, 4b). Similarly, for the HER2-positive subtype (PFS 7.2 m vs. 13.7 m, *p* < 0.001; OS 21.7 m vs 26.2 m, *p* = 0.048; Figs. 3c, 4c) and luminal subtype (PFS 8.0 m vs. 12.3 m, *p* = 0.026; OS 21.9 m vs 29.9 m, *p* = 0.021; Figs. 3d, 4d) breast cancers, high sPD-L1 level remained significantly associated with poor PFS and OS.

Univariate analysis and multivariate analysis identified a high sPD-L1 level and visceral metastasis as factors associated with poor prognosis (Table 2). A high plasma sPD-L1 level (≥ 8.774 ng/ml) was an independent risk factor that more significantly affected PFS (HR = 3.358, 95%CI: 2.425–4.650, *p* < 0.001) and OS (HR = 2.792, 95%CI: 1.863–4.184, *p* < 0.001).

**Table 2:** Univariate and multivariate Cox analyses for mPFS and mOS

	mPFS						mOS					
	Univariate analysis			Multivariate analysis			Univariate analysis			Multivariate analysis		
	HR	95%CI	*p* value	HR	95%CI	*p* value	HR	95%CI	*p* value	HR	95%CI	*p* value
*Age,years*												
≤ 60 vs > 60	1.178	0.181–1.712	0.384				0.930	0.568–1.525	0.930			
*Menopausal status*												
Pre- vs postmenopausal	1.170	0.861–1.589	0.316				0.989	0.664–1.472	0.956			
*IHC profile*												
Triple-negative vs Luminal	1.059	0.661–1.699	0.811				1.128	0.846–1.504	0.411			
HER2-positive vs Luminal	1.030	0.735–1.443	0.863				0.980	0.633–1.518	0.929			
*Location of metastases*												
Visceral vs non-visceral	**2.728**	**1.862–3.997**	** < 0.001**	**3.786**	**2.545** **–5.633**	** < 0.001**	**4.066**	**2.113–7.826**	** < 0.001**	**4.976**	**2.575–9.618**	** < 0.001**
*Postoperative TNM staging*												
I-II vs III	0.763	0.550–1.058	0.104				0.836	0.552–1.266	0.396			
*Previous chemotherapy*												
Yes vs No	1.359	0.795–2.322	0.262				0.980	0.520–1.844	0.949			
*Previous endocrine therapy*												
Yes vs No	0.973	0.702–1.349	0.871				0.812	0.536–1.230	0.325			
*Previous radiotherapy*												
Yes vs No	1.264	0.894–1.786	0.185				1.507	0.944–2.405	0.086			
*Plasma sPD-L1 level*												
≥ 8,774 vs <8.774	**2.342**	**1.720–3.189**	** < 0.001**	**3.358**	**2.425–** **4.650**	** < 0.001**	**2.286**	**1.530–3.415**	** < 0.001**	**2.792**	**1.863–4.184**	** < 0.001**

Abbreviations: sPD-L1, soluble programmed death-ligand 1; IHC, immunohistochemical; HR, hazard ratio; CI, confidence interval; mPFS, metastatic progression-free survival

Bold values indicate univariate and multivariate analyses identified high sPD-L1 level (≥ 8.774 ng/ml) and visceral metastasis were independent factors associated with poor prognosis

### Decreased sPD-L1 level in partial remission patients

All of the recurrent/metastatic patients received rescue first-line treatment in accordance with patient preference, tumor biology and disease clinical features. A total of 99 (47.6%) HER2-positive metastatic patients received trastuzumab plus taxane chemotherapy or lapatinib plus capecitabine chemotherapy. A total of 27 (13.0%) HER2-negative luminal metastatic patients received aromatase inhibitors (AIs) combined medicine or surgical deprivation therapy or fulvestrant therapy. A total of 82 (39.4%) received capecitabine monotherapy, gemcitabine monotherapy or combined platinum, vinorelbine monotherapy or combined platinum, and taxane monotherapy or taxane-based combined therapy.

Of these, 32 patients achieved partial remission (PR) according to RESIST criteria version 1.1. The plasma sPD-L1 levels of these patients were found to significantly decrease compared with their corresponding levels in plasma collected before first-line treatment (*p* = 0.049, Fig. 1c). Nevertheless, 11 (34.4%) of the 32 patients showed no disease progression at the time of the last follow-up (median mPFS: 15.0 m).

### Correlation between tumoral PD-L1 and sPD-L1 level in breast cancer patients

**Fig. 5:**
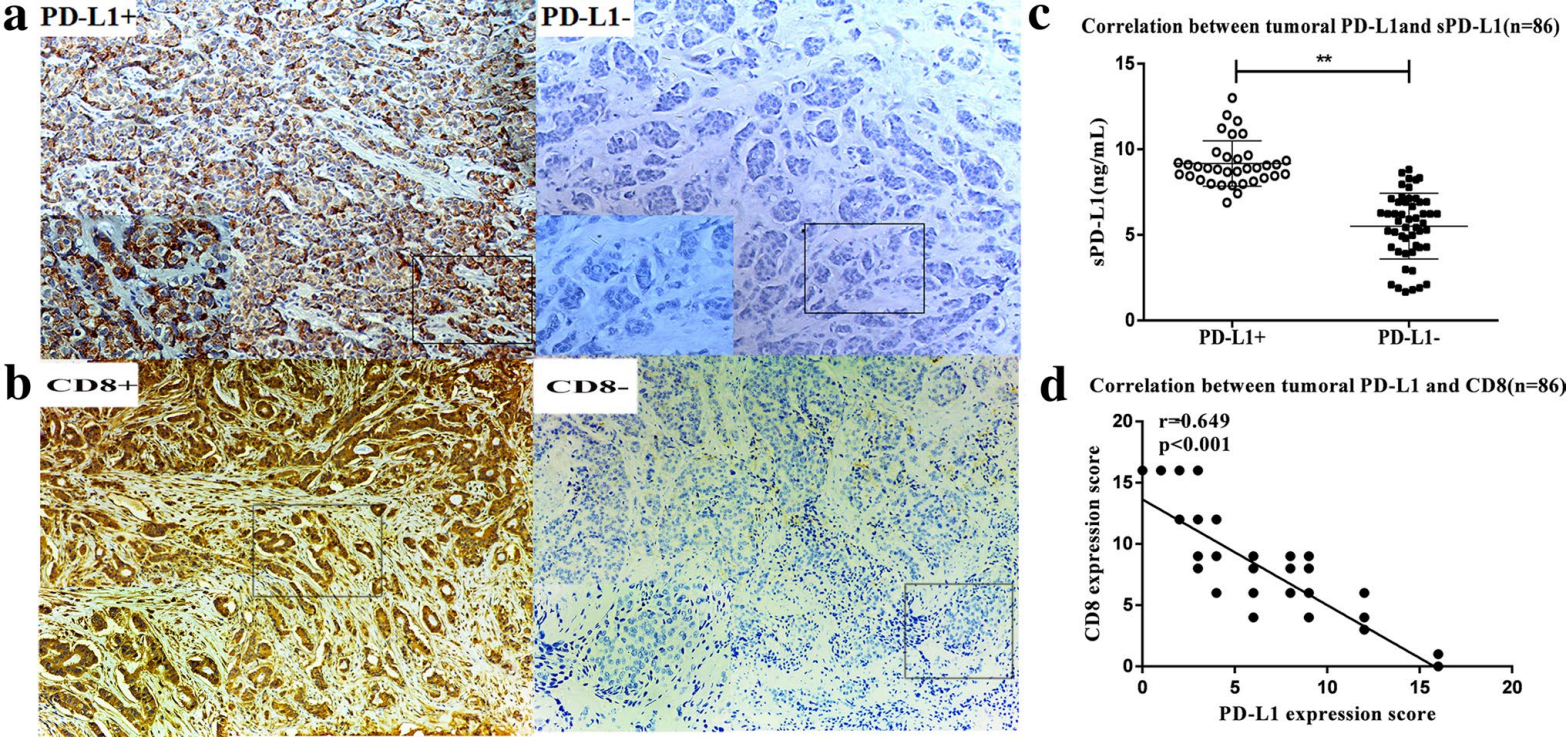
Immunohistochemical staining of tumoral PD-L1 expression and correlation with corresponding plasma sPD-L1 level. **a** Immunohistochemical staining of PD-L1 expression in advanced breast cancer (10 × HPF and 40 × HPF), PD-L1 staining in ≥ 1% of tumor cells or in stroma cells(left), PD-L1 staining in < 1% of tumor cells (right). **b** Immunohistochemical staining of CD8 expression in advanced breast cancer (10 × HPF and 40 × HPF), PD-L1 staining in ≥ 1% of tumor cells or in stroma cells(left), PD-L1 staining in < 1% of tumor cells (right). **c** Correlation between tumoral PD-L1 expression and corresponding plasma sPD-L1 level (*n* = 86). Statistical analysis was performed using the Mann–Whitney U test. **, *p* < 0.01. **d** A statistically significant inverse correlation between tumoral PD-L1 and CD8 expression score in 86 cases of breast cancer tissues

The direct comparison of the clinical burden between tissue and serum sPD-L1 had not been examined. Of the 86 patients with recurrent/metastatic breast cancer whose tumor samples were screened for PD-L1 expression (77 in nodal and soft tissue, 9 in liver tissue), 35 (40.7%) presented PD-L1 expression in stroma or at least 1% of tumor cells (as shown in Fig. 5a). In the analysis of tumoral PD-L1 expression and the corresponding plasma sPD-L1 level, a significant correlation was observed (*p* < 0.01, Fig. 5c).

### Immunohistochemical analysis of CD8-positive T cells

We evaluated the correlation between the numbers of CD8-positive T cells and tissue PD-L1 expression by IHC (Fig. 4a, b). We found CD8-positive T cells were significantly decreased with elevated tissue PD-L1 expression in the breast cancer tissues (Fig. 5d).

### Correlation between TILD and sPD-L1 level in breast cancer patients

We analyzed the correlation between TILD (tumor-infiltrating lymphocyte density) and sPD-L1 level in breast cancer patients. Of the 86 patients with recurrent/metastatic breast cancer whose tumor samples were screened for TILD expression, 50 (58.1%) presented TILD expression at least 20% (as shown in (Supplementary Fig. 2). In the analysis of TILD expression and the corresponding plasma sPD-L1 level, a significant correlation was observed (*p* < 0.001, Supplementary Fig. 2).

### sPD-L1 expression in supernatant of breast cancer cells

**Fig. 6:**
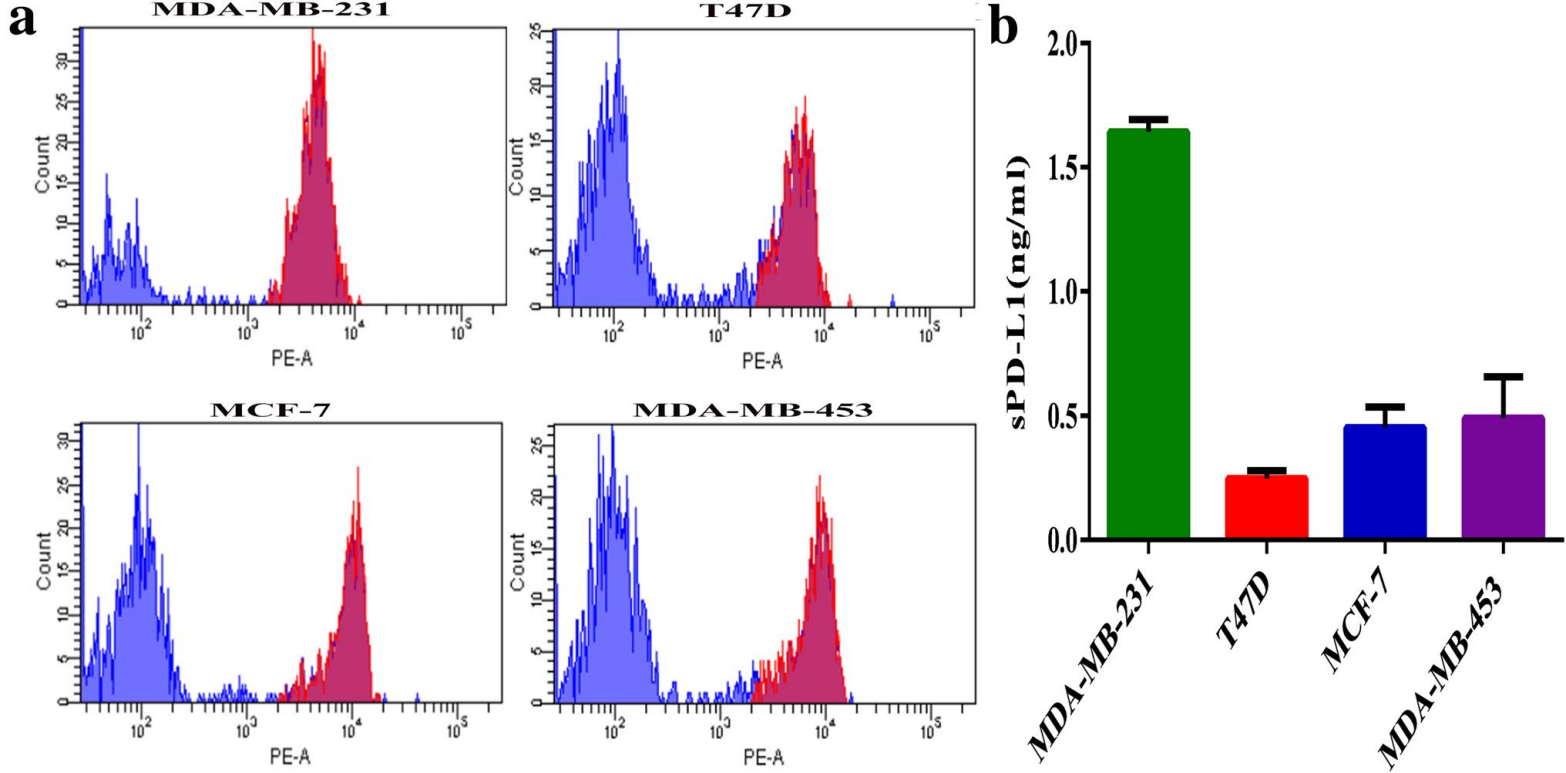
sPD-L1 expression in supernatant of breast cancer cells: **a** flow cytometry was analyzed with mPD-L1 expression on breast cancer cells. **b** The cell-free supernatants of MDA-MB-231, T47D, MCF-7 and MDA-MB-453 cells were collected to determine the levels of sPD-L1 by the ELISA method

To reveal whether breast cancer cell lines expressing mPD-L1 can produce soluble PD-L1, PD-L1 expression on breast cancer cells was analyzed by flow cytometry and the level of sPD-L1 in the supernatant of breast cancer cells was determined with an ELISA kit (PDCD1LG1 ELISA kit, USCN Life Science, Wuhan, China). It showed that sPD-L1 could be detected in the supernatant of the culture of mPD-L1 ( +) breast cancer cell lines (Fig. 6a, b).

### sPD-L1 inhibits the proliferation of T lymphocytes

**Fig. 7:**
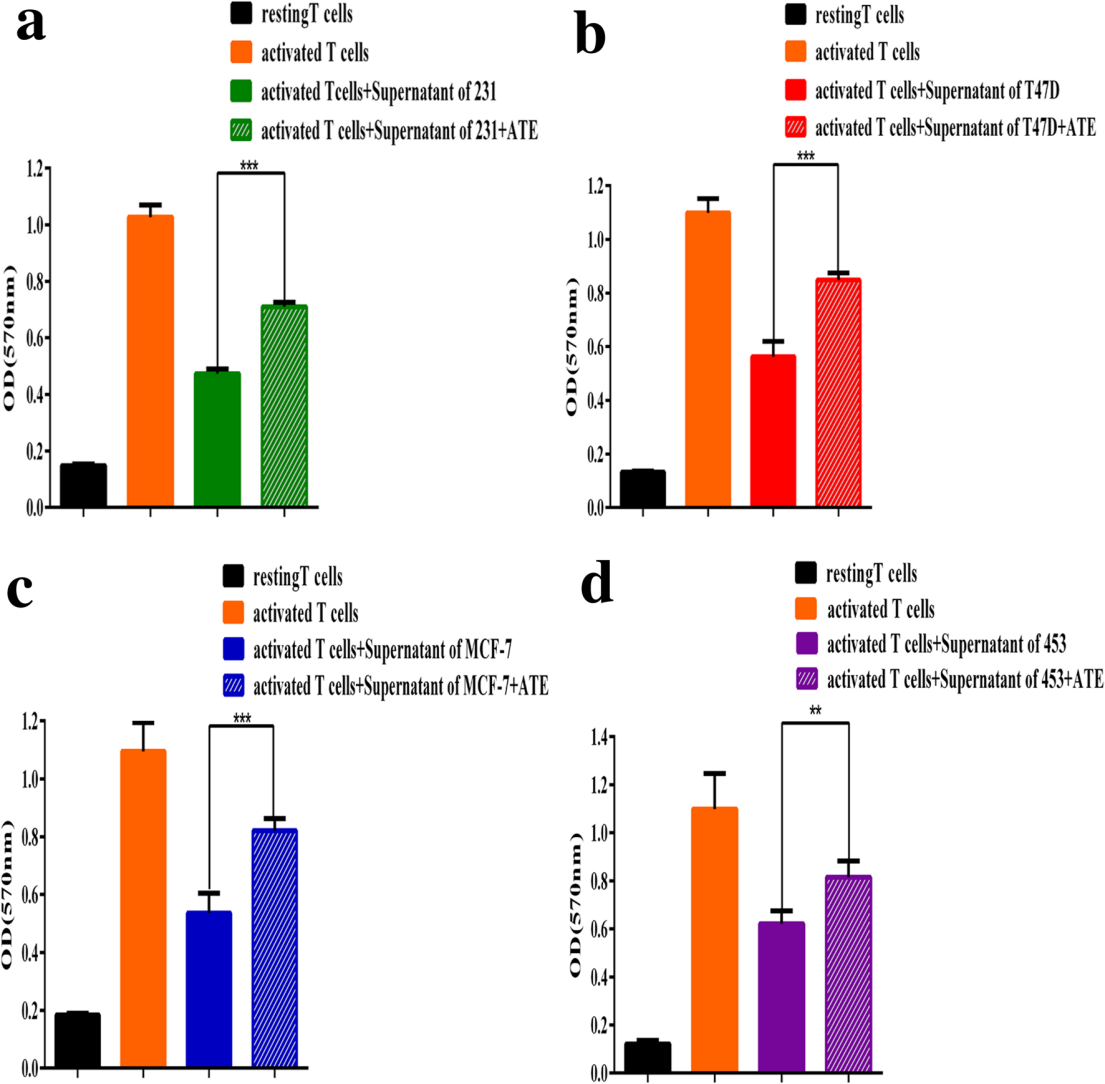
sPD-L1 inhibits proliferation of T lymphocytes. **a–d** WST-1 analysis of cell proliferation T lymphocyte when it was co-cultured with 231, T47D, MCF-7 and 453 cells or ATE (anti-PD-1 mAb (100 μg/ml)). **, *p* < 0.01; ***, *p* < 0.001. These data are representative of three independent experiments

We next assessed the effect of the inhibition of T lymphocyte after co-culture with the supernatant of MDA-MB-231, T47D, MCF-7, MDA-MB-453 breast cancer cell lines expressing sPD-L1 by WST-1. T cell proliferation was inhibited by co-culture with MDA-MB-231, T47D, MCF-7, and MDA-MB453 breast cancer cell lines producing sPD-L1, whereas sPD-L1 could effectively restore the inhibitory effect of PD-1/PD-L1 on T lymphocytes after the addition of anti-PD-L1 antibody (atezolizumab). The results showed that sPD-L1 could inhibit the proliferation of T lymphocytes (*p* < 0.001, *p* < 0.001, *p* < 0.001 and *p* < 0.01, respectively, Fig. 7a–d).

### sPD-L1 increases the apoptosis rate of T lymphocytes

**Fig. 8:**
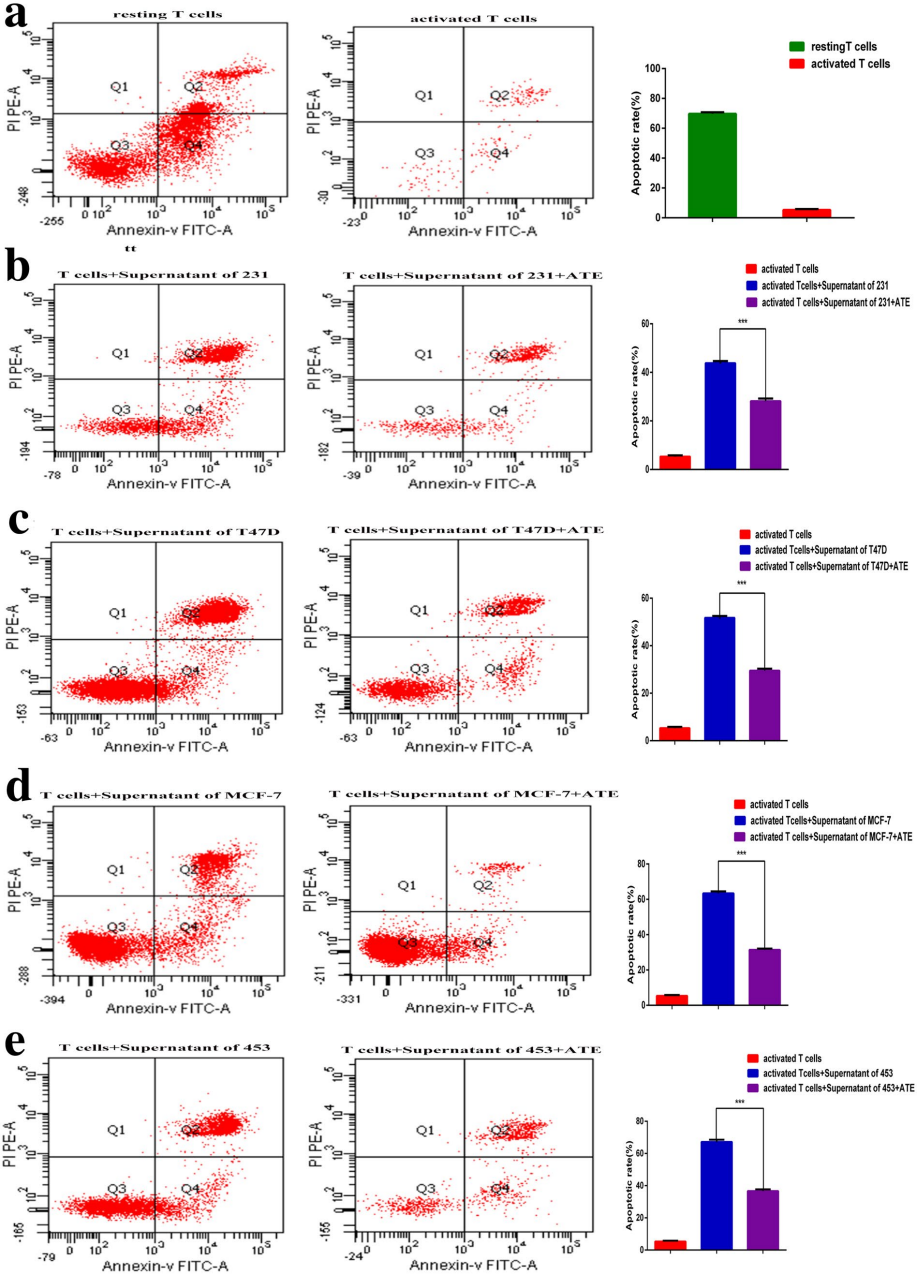
sPD-L1 increase the apoptosis rate of T lymphocytes. **a–e** Flow cytometric analysis of T cell apoptosis in 231, T47D, MCF-7 and 453 cells, in which cancer cells were co-cultured with activated T cells alone or activated T cells with ATE at 100 µg/ml concentrations for 72 h time; ***, *p* < 0.001

We performed flow cytometry assays to investigate the effect of sPD-L1 on regulating the apoptosis of T lymphocytes. Compared with the group without anti-PD-L1 antibody, the apoptosis rate of containing anti-PD-L1 antibody group was significantly decreased, the same results were observed in MDA-MB-231, T47D, MCF-7, MDA-MB-453 breast cancer cell lines (*p* < 0.001 all in Fig. 8a–e). We found that sPD-L1 could promote apoptosis of activated T lymphocytes, whereas the effect could also be reversed by the adding of antibody against PD-L1.

## Discussion

Recent studies indicated that cancer-associated fibroblasts (CAFs), tumor-associated macrophages (TAMs) and regulatory T cells (Tregs) within the tumor microenvironment were involved in the process of breast cancer growth, invasion and metastasis [26–28]. Immune checkpoint molecules such as PD-1, PD-L1 and CTLA4 also play a pivotal role in immunosuppression of the tumor microenvironment [6]. Immunotherapy with immune checkpoint inhibitors is a promising and rapidly growing field of interest in many solid tumors; to date, pembrolizumab and atezolizumab, two checkpoint inhibitors, have been most extensively studied in breast cancer [29–33].

Except for the expression of immune checkpoint molecules in the tissues, sPD-L1 detection also drew attention. Recently, research has shown that high sPD-L1 levels were proved as prognostic for poor treatment response and survival prognosis in hepatocellular carcinoma [34], renal cell cancer [35], ovarian cancer [36], lung cancer [37], gastric cancer [38], melanoma [39] and extranodal NK/T cell lymphoma [40]. To the best of our knowledge, the current work is the first to investigate the clinical prognostic complications of plasma sPD-L1 in recurrent or metastatic breast cancer patients before receiving first-line rescue therapy. In our study, it suggested that the plasma sPD-L1 level was comparatively higher in recurrent/metastatic patients than early-stage patients.

Several studies have shown that PD-1/PD-L1 play an important role in the occurrence and development of breast cancer. IHC studies showed that PD-L1 expression in breast cancer was an unfavorable indicator associated with poor DFS and OS [41]. A genomic analysis on mRNA expression of immune regulatory molecules in PBMCs from primary and metastatic breast cancer patients suggested a correlation between PD-L1 gene and disease progression [23]. In our study, we found that sPD-L1 level greater than 8.774 ng/ml measured in the peripheral blood before first-line treatment was significantly associated with a short PFS of recurrent or metastatic breast cancer. Consistent with a report in the 2017 ASCO meeting that higher serum sPD-L1 levels were prognostic for poor PFS and OS in HER2-positive metastatic breast cancer patients treated with first-line trastuzumab. Luminal-like subtypes patients corresponded to 36.5% of our cohort, which would get a long survival if they received an useful treatment regimen. Hence, we did not compare the correlation between sPD-L1 levels and OS of these breast cancer patients. Because breast cancer was a heterogeneous disease, different subtypes have different molecular and immunohistochemical profiles, prognoses, and responses to treatment [29–32]. However, in our study, plasma sPD-L1 expression showed no difference in different subgroups of breast cancer (triple-negative, HER2-positive, and luminal-like).

Good tumor biomarkers were expected to predict the therapeutic effect of anti-tumor drugs. A clinical trial reported that a significant decrease of the sPD-L1 level in DLBCL patients achieved CR of at least half year compared with the corresponding levels at diagnosis [19]. Additionally, we found that elevated levels of sPD-L1 were associated with poorer prognosis, regardless of the assigned treatment, and that sPD-L1 levels decreased dramatically for patients who achieved PR, especially those with high sPD-L1 levels. These results indicated that sPD-L1 might be representative of an anti-tumor immunosuppression state and an improvement in intrinsic adaptive anti-tumor immunity via effective therapy. These results cleared the way for the further studies aimed at determining whether monitoring the alternation of sPD-L1 level before and after therapy in the peripheral blood can identify patients who are most likely to benefit from conventional treatments.

Circulating inflammatory cells, including lymphocytes, monocytes, neutrophils, and their combined index (LMR, NLR), reflect a systemic inflammatory response to cancer [42], which indicates that systemic inflammatory response is an important prognostic factor in tumor development and progression [43]. Lymphocytes inhibit tumor cell proliferation and migration, induce cytotoxic cell death and subsequently eradicate cancer, and in contrast, monocytes release cytokines and free radicals related to angiogenesis, tumor growth and distant spread. Neutrophils mediate tissue damage via certain biochemical mechanisms such as the release of arachidonic acid metabolites, oxidative free radicals and other hydrolytic enzymes. The current study shows significant relationships between selected inflammatory markers (monocyte count, neutrophil count) and plasma sPD-L1 levels. He et al. found that the ratio of PD-L1( +) neutrophils to PD-1( +) T cells was higher in peritumoral tissue and better predicted the disease-free survival of patients with HCC [42]. Compared with healthy subjects, interferon-γ, IL-6 and IL-10 were significantly increased in the plasma of high-sPD-L1 patients [44]. These data support the hypothesis that sPD-L1 could indicate the anti-immune response of the disease and cooperatively impact tumor progression.

Furthermore, findings also showed that soluble PD-L1 associated immune suppression via regulation the function of T lymphocyte. Shi B. et al. [45] stated that sPD-L1 may contribute to the proliferation of T cells and the development of diabetic macrovascular diseases. Pan X. et al. [46] suggested that the immune mechanism of sPD-L1 and the PD-1/PD-L1 pathway is associated with immune response in tuberculous pleural effusion. Avendaño-Ortiz J. et al. [47] demonstrated that elevated concentration of sPD-L1 in sepsis results in the lowest rate of T cell proliferation by PD-L1/PD-1 cross talk. Blinova E. et al. [48] showed negative correlation of sPD-L1 serum concentration and CD8 + tumor expression in subgroups of Durvalumab-treated mice that carried both primary and relapsed non-muscular invasive bladder cancer of GATA 3 and KRT 5/6 expressed subtypes. Wu D. et al. [49] reported that activation of the PD-1/PD-L1 pathway using sPD-L1 could improve the imbalance of Th1/Th2 and Treg/Th17 immune cells in ITP patients. Li Y. et al. [50] stated that by controlling the expression of sPD-L1, it may be possible to block the inhibitory effect of the PD-1/PD-L1 signaling pathway and improve the function of effector T cells in cystic echinococcosis. Orme et al. [51] showed that sPD-L1 from tumor cells induces CD8 + T cell death and inhibits anti-tumor immunity. Although sPD-Ll have been recognized as naturally existing regulators of PD-1/PD-L1 membrane signaling pathways in various disease systems, the biological activity of sPD-L1 remains incompletely understood, clearly, further work is needed to better understand the implications of sPD-L1 levels on immunotherapy efficacy in breast cancer. The results of this study indicate that, like other soluble factors with immunoregulatory functions, sPD-L1 was present in a functional form in breast cancer cells. In vitro, MDA-MB-231, T47D, MCF-7, MDA-MB-453 breast cancer cell lines produced sPD-L1, the supernatant of breast cancer cells containing sPD-L1 significantly inhibited the proliferation of PHA-stimulated T cells. Atezolizumab blocked the interaction of PD-1/sPD-L1 and effectively restored the proliferative capacity of T cells. It can be speculated that with the increased of sPD-L1, more sPD-L1 bound to PD-1 on the surface of activated lymphocytes, which limited the activation and proliferation of T lymphocytes, leading to the killing effect of breast cancer cells on immune cells [37]. Studies had shown that the expression of soluble costimulatory molecules was significantly correlated with clinicopathological features such as lymph node metastasis, tumor size and multiple organ metastasis.

To summarize, sPD-L1 is a good tumor maker in recurrent or metastatic breast cancer patients before receiving first-line rescue therapy, and high plasma levels of sPD-L1 are associated with a shorter PFS. This is the first study to confirm the negative impact of sPD-L1 on the progress of recurrent or metastatic breast cancer in the patient population. Abnormality of soluble factors plays an important role in the long-term and early immune regulation of the body, and it is beneficial to the tumor cells to resist the killing and elimination of lymphocytes in the tumor microenvironment. Specific anti-PD-L1 antibody can reduce the expression of sPD-L1 and remove its blocking effect on PD-1/PD-L1 negative signaling pathway, which may help to improve T cell viability and enhance the killing for breast cancer cells.

## Supplementary information

Below is the link to the electronic supplementary material.

ROC curve analysis for the optimal cutoff value of plasma sPD-L1 concentration. (TIF 11906 KB)

Correlation between TILD and sPD-L1 level in breast cancer patients, ***, p<0.001. (TIF 345630 KB)

Supplementary file3 (DOCX 14 KB)

## Abbreviations

Ais: aromatase inhibitors
CAFs: cancer-associated fibroblasts
CEA: carcinoembryonic antigen
CI: confidence interval
CR: complete remission
DFS: disease-free survival
DLBCL: diffuse large B cell lymphoma
ELISA: enzyme-linked immunosorbent assay
HR: hazard ratio
IHC: immunohistochemistry
NE: non-evaluable
OS: overall survival
PBMCs: peripheral blood mononuclear cells
PD: disease progression
PFS: progression-free survival
PR: partial remission
RECIST: Response Evaluation Criteria in Solid Tumors
SD: stable disease
sPD-L1: soluble programmed cell death ligand 1
TAMs: tumor-associated macrophages
TILD: tumor-infiltrating lymphocyte density
Tregs: regulatory T cells
