# Supplementary figures and images for: The clinical implication of soluble PD-L1 (sPD-L1) in patients with breast cancer and its biological function in regulating the function of T lymphocyte
Source: Cancer Immunol Immunother. 2021 Mar 10;70(10):2893–909. doi: 10.1007/s00262-021-02898-4 (PMC8423647; doi:10.1007/s00262-021-02898-4)

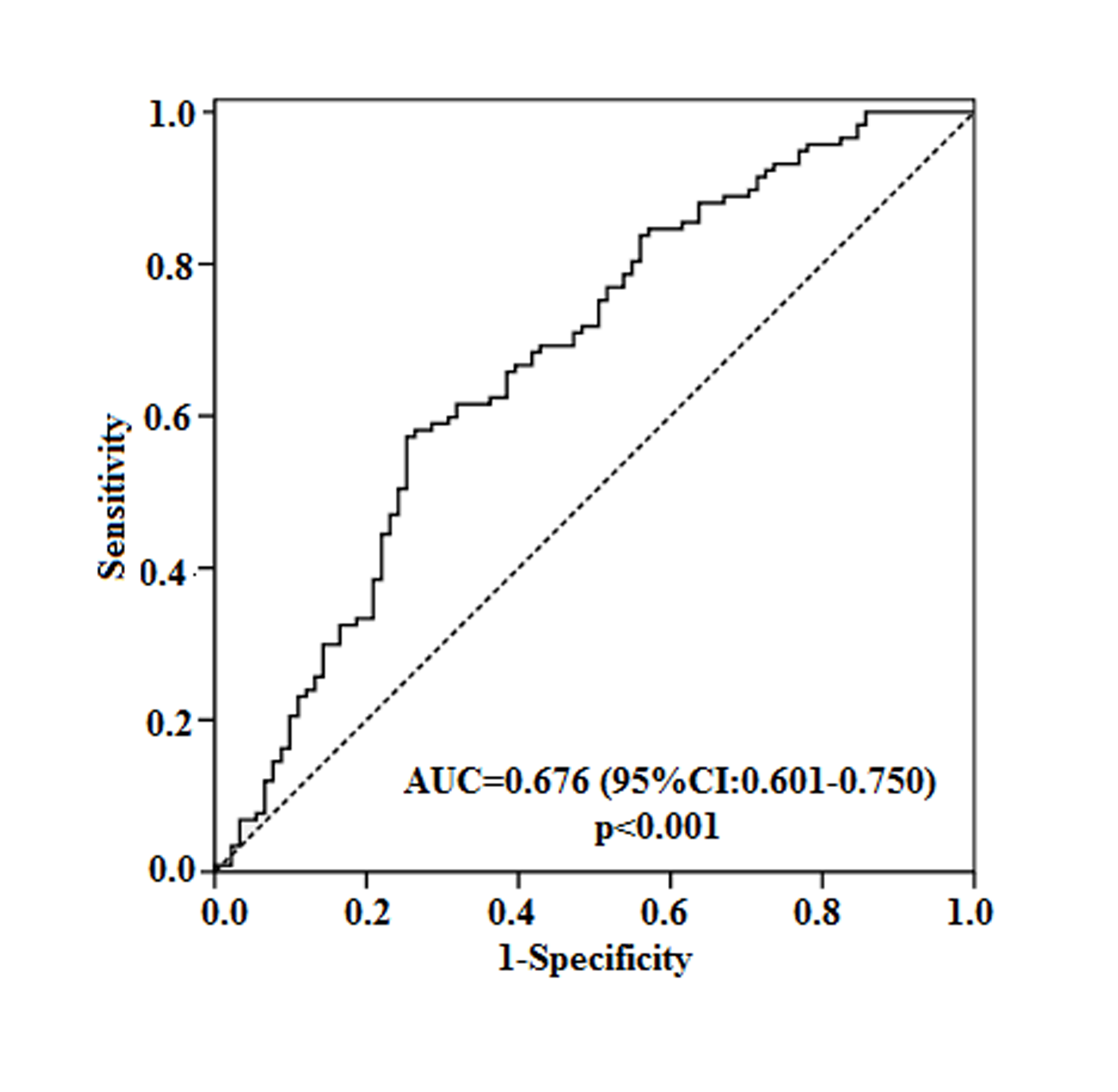

Supplement: Supplementary file 1 — ROC curve analysis for the optimal cutoff value of plasma sPD-L1 concentration. (TIF 11906 KB) [file 262_2021_2898_MOESM1_ESM.tif]
