# Supplementary material for: The clinical implication of soluble PD-L1 (sPD-L1) in patients with breast cancer and its biological function in regulating the function of T lymphocyte
Source: Cancer Immunol Immunother. 2021 Mar 10;70(10):2893–909. doi: 10.1007/s00262-021-02898-4 (PMC8423647; doi:10.1007/s00262-021-02898-4)
Supplement: Supplementary file 3 — Supplementary file3 (DOCX 14 KB) [file 262_2021_2898_MOESM3_ESM.docx]

**Table S1. Clinical characteristics of early breast cancer patients**

| **Characteristics** | **n of patients** | **%** |
| --- | --- | --- |
| **Age, yrs** |  |  |
| ≤ 60 | 22 | 68.8 |
| > 60 | 10 | 32.2 |
| **IHC profile** |  |  |
| Triple-negative | 5 | 15.6 |
| HER-2-positive | 15 | 46.9 |
| Luminal | 12 | 37.5 |
| **Menopause status** |  |  |
| Premenopausal | 16 | 50.0 |
| Postmenopausal | 16 | 50.0 |
| **TNM staging** |  |  |
| I-II | 19 | 59.4 |
| III | 13 | 40.6 |

IHC, immunohistochemical
